# Impact of Volume-Oriented Incentive Spirometry on Lung Volume and Peak Expiratory Flow Rate in Patients With Tracheostomy

**DOI:** 10.7759/cureus.56820

**Published:** 2024-03-24

**Authors:** Arumugam Sankar Ganesh, Kumaresan Abathsagayam, Natesh Prabu Ravisankar

**Affiliations:** 1 Department of Physiotherapy, Saveetha College of Physiotherapy, Saveetha Institute of Medical and Technical Sciences, Chennai, IND; 2 Department of Critical Care Medicine, St. John's Medical College Hospital, Bengaluru, IND

**Keywords:** respiratory rehabilitation, peak expiratory flow rate, lung volume, tracheostomy, incentive spirometry

## Abstract

Background

The volume-oriented incentive spirometer is a specialized device designed to facilitate maximal inspiration, promote deep breathing exercises, and enhance lung function. The use of spirometry is challenging and not proven in patients with tracheostomy. Therefore, this study aimed to assess the impact of volume-oriented incentive spirometry on lung volume and peak expiratory flow rate (PEFR) in patients with tracheostomy.

Methodology

All adult patients with cuffed tracheostomy tubes with a Medical Research Council (MRC) score of more than 48 were studied. Volume-oriented incentive spirometry was performed and the PEFR was measured before and after the spirometry session. All patients underwent 28 sessions in seven days with initial few training sessions. Patient demographic information, such as age, gender, reasons for tracheostomy, MRC at the beginning of the session, volume (volume per breath, mL), and PEFR, was documented.

Results

Thirty patients were studied, consisting of 18 males and 12 females with initial MRC scores ranging from 48 to 60. The mean lung volume and mean PEFR at the end of seven days were 950 ± 330.9 and 134.7 ± 63.3, respectively, demonstrating safety with minimal complications, including four cases of pain at the tracheostomy site, three cases of hypotension, one case of abdominal pain, and 22 cases with no reported complications.

Conclusion

Volume-oriented incentive spirometry improves lung volume and PEFR in patients with a tracheostomy tube. Additionally, spirometry proved to be both feasible and effective in this patient population.

## Introduction

An incentive spirometer serves as a specialized device engineered to facilitate and sustain maximal inspiration by actively engaging the diaphragm and other crucial inspiratory muscles. Its primary function involves encouraging individuals to perform deep inspiratory and expiratory breathing exercises characterized by long, profound breaths followed by deliberate pauses [1,2]. This technique emphasizes lung inflation, fostering an increase in tidal volume, and upholding the opening and functionality of smaller airways within the respiratory system [1].

For patients undergoing chest physiotherapy, integrating incentive spirometry into their respiratory care regimen is strongly recommended [3]. Incentive spirometry plays a pivotal role in this context by actively engaging patients in their recovery process. By encouraging active participation, the device empowers patients to take an active role in their respiratory rehabilitation, promoting a sense of ownership and involvement in their recovery journey [4,5].

The application of incentive spirometry in individuals who have undergone tracheostomy remains an understudied area within existing medical research. Patients who have undergone tracheostomy represent a unique subset with distinct health challenges, particularly with increased vulnerability to pulmonary complications [6-8]. Several contributing factors exacerbate this susceptibility, including prolonged periods of immobility, extended stays in the intensive care unit, compromised nutritional status, and a significant history of tobacco use, often coupled with underlying chronic pulmonary conditions. These collective factors significantly heighten the risk of pulmonary complications among individuals with tracheostomy [4,9].

By incorporating incentive spirometry into the care plan for individuals with a tracheostomy, healthcare providers can enhance respiratory therapy outcomes, facilitate better lung expansion, promote airway clearance, and ultimately reduce the risk of respiratory complications, thereby contributing to a smoother recovery process and improve overall pulmonary health for these patients [4,10].

However, despite the beneficial aspects of conventional spirometers in promoting optimal respiratory function, their use in patients with a tracheostomy tube is currently limited. This limitation arises from the inability to connect the spirometer's mouthpiece directly to the tracheostomy tube, which prevents seamless integration of the device into the breathing regimen of these individuals [11]. Consequently, the efficacy of such spirometers remains uncertain in patients with tracheostomies. Notably, patients with a tracheostomy may not sustain the intrathoracic pressure since the vocal cords are bypassed by tracheostomy and this, in turn, may affect the effective use of the spirometer. This challenge arises from the fact that the tracheostomy tube bypasses the vocal cords, impacting the patient's ability to generate and sustain the necessary pressure for optimal use of the spirometer [12,13].

Therefore, the current landscape of available spirometers presents limitations in their suitability for patients with tracheostomies, both in terms of physical compatibility and uncertainties regarding their effectiveness in this specific patient population [4,14,15]. This underscores the need for innovative spirometry designs or alternative respiratory interventions tailored specifically for individuals with tracheostomies to address these challenges and optimize respiratory therapy for this unique patient group [12,16]. Additionally, previous studies have not addressed whether such device use is effective in improving lung function after the recommended session of spirometry, which is an important outcome measure after spirometry. Increases in the volume of air displaced with patient effort, improvement in activities of daily living, or measuring peak expiratory flow rate (PEFR) are commonly used to assess the improvement of respiratory function [3,4,17].

An increase in the volume of air displaced with patient effort is easy to measure and is assessed at the bedside. PEFR is a simple and non-invasive measure that relies on voluntary effort, expiratory muscle strength, airway resistance, and lung elastic recoil. This low-cost device is effective in measuring flows from 60 to 800 L/min. However, normative values are typically available only for patients without tracheostomy who breathe normally [18,19]. To adapt for tracheostomy patients with a cuffed tube, adjustments to the PEFR device's mouthpiece are essential and can be easily achieved using a short connector to the tracheostomy tube similar to the connector used by Malhotra et al. [3] and Goldstein et al. [4] to check the feasibility of spirometry in patients with tracheostomy tube [20].

Recognizing this critical gap in research and the dire need to address the respiratory health of this specific patient cohort, the objective of the present study was to assess the impact of volume-oriented incentive spirometry on lung volume and PEFR in patients with tracheostomy, as understanding how this intervention influences lung volume and PEFR in patients with tracheostomy holds significant promise in improving their respiratory outcomes and potentially mitigating the risk of associated pulmonary complications.

## Materials and methods

This prospective study was conducted at a tertiary care teaching hospital. The study was approved by the Institutional Ethical Committee with reference number ST-222/2022 and registered with the Clinical Trials Registration of India with reference number CTRI/2022/12/048599. The study included patients from January 2023 to November 2023. The inclusion criteria involved all adult patients with a cuffed tracheostomy tube admitted to the hospital, with a Medical Research Council (MRC) scale score of more than 48, and who were willing and consented to participate. Whereas pregnant or lactating patients, patients having rib fractures, patients demonstrating non-cooperative behavior, and patients showing an unwillingness to participate, or an inability to comprehend study procedures were excluded from the study.

**Figure 1:**
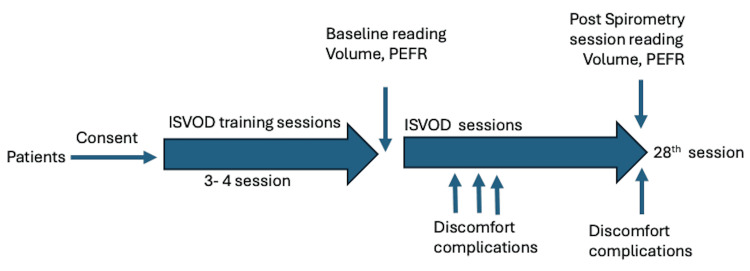
Study procedure ISVOD = volume-oriented incentive spirometer device; PEFR = peak expiratory flow rate.

Based on the inclusion and exclusion criteria, a total of 30 patients were included in the present study. Patient-specific data encompassing demographic information, such as age, sex, the reason for tracheostomy, and MRC score, were recorded. All patients were instructed and demonstrated about incentive spirometry and PEFR. To ensure proficiency in the volume-oriented incentive spirometer device (ISVOD) technique, all subjects underwent comprehensive training sessions where they were assessed on how they performed, the correctness of the technique, their comfort, and complications as mentioned below. The initial three sessions of a total of 28 sessions were primarily dedicated to instructing and familiarizing patients with the correct approach and technique for ISVOD. However, recognizing individual learning curves and variations in technique adoption, additional sessions beyond the initial three were facilitated whenever necessary. These extra sessions were aimed at ensuring that patients adapted to employing the correct technique consistently, thereby optimizing the benefits derived from ISVOD. Figure 1 demonstrates the study procedure followed in this study.

The exercises with volume-oriented devices were performed as per the guidelines of the American Association for Respiratory Care [21], which recommends that individuals inhale slowly and deeply, hold their breath at maximal inspiration for at least three seconds, and exhale normally. The participants followed these rules and a diagram was used to instruct them on how to perform the exercises. The volume-oriented device used was the Spiro Ball Spirometer Coach 2 device (MediMark Europe, Grenoble, France). Patients were directed to relax their upper chest, shoulders, and arms while focusing on engaging the lower chest and abdomen during a deep inhalation. This technique aimed to maximize the effectiveness of ISVOD by emphasizing the expansion of the lower chest and abdomen while minimizing unnecessary tension in the upper body regions [22,23]. Patients were asked to make an inspiratory effort and sustain it for a duration of three to five seconds. This was repeated for 10 successive cycles with a 30-second break between breaths, which constituted one session. This method was carried out during the patient’s waking hours. Four sessions were performed per day, a total of 28 sessions over seven days.

**Figure 2:**
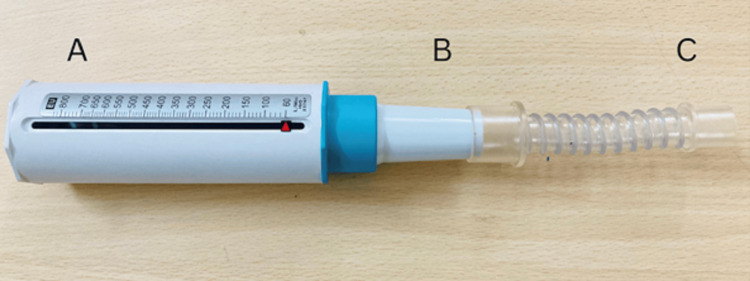
Parts of the customized peak expiratory flow rate meter for patients with a tracheostomy tube A = conventional peak expiratory flow rate (liter per minute); B = mouthpiece; C = short connector (gooseneck tubing).

PEFR (Breathe O Meter, Cipla Ltd, Mumbai, India) was measured using a short connector attached to the cuffed tracheostomy tube and peak expiratory flow meter, as shown in Figure 2. Similarly, subjects were directed to relax before conducting the PEFR measurement. The testing procedure was thoroughly explained to each subject. Following a period of rest, subjects were guided to inhale deeply and then exhale forcefully in one continuous breath into the instrument. Three acceptable readings were recorded, and the highest value among the three was considered. Vigilance was maintained to ensure a secure seal between the tracheostomy and the PEFR device throughout the measurement process. The PEFR value was recorded with precision and rounded to the nearest liter per minute.

**Figure 3:**
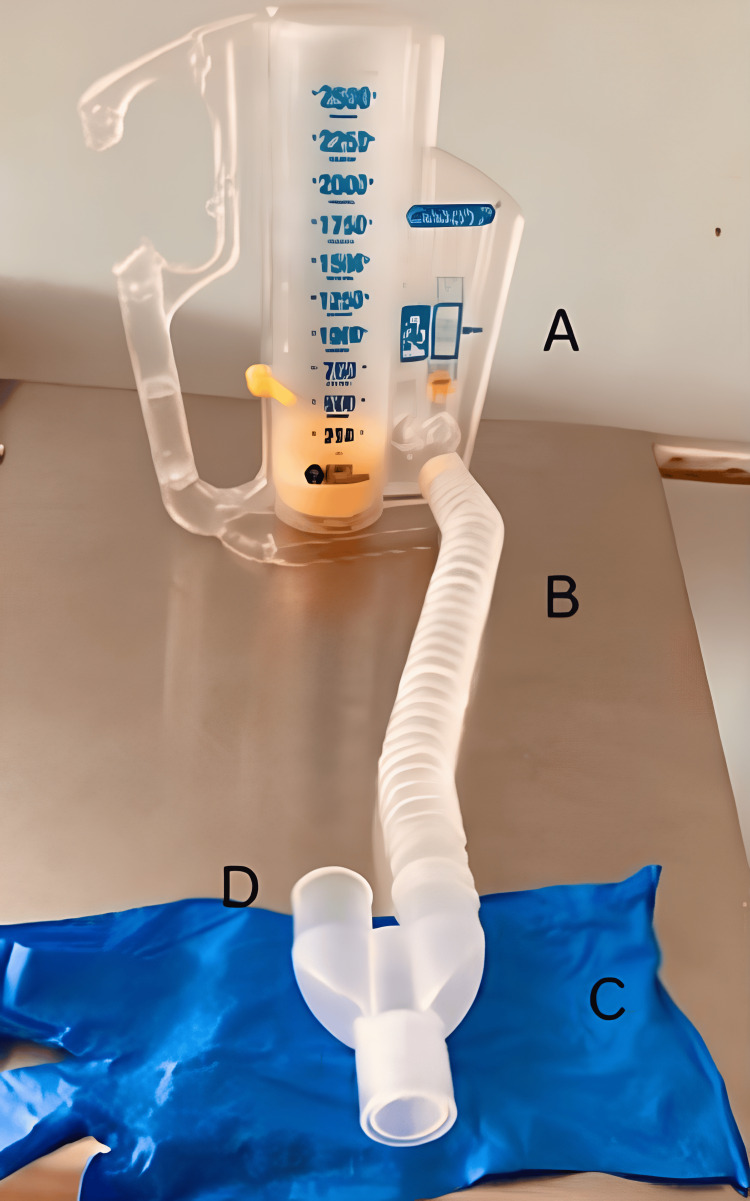
Parts of the customized incentive-oriented spirometer modified for patients with a tracheostomy tube A = conventional volume-oriented incentive spirometer; B = gooseneck tubing; C = wye adapter; D = provides an extra safety measure by providing an open connection. The opening is occluded manually with a finger during inspiration and released/opened during expiration to facilitate expiration.

The volume-oriented spirometer modified for patients with a tracheostomy tube used for the present study is shown in Figure 3. The spirometer’s wye adapter design is an altered rendition of the model delineated in the study given by Goldstein et al. [4]. The wye adapter, bearing a ‘Y’ form, comprises two arms and a single stem. The stem was connected to the standard tracheostomy tube with an external diameter of 12.0 mm, and one arm was connected to the incentive spirometer’s mouthpiece part using a gooseneck tube provided with the device. The other arm is left open and the therapist can readily occlude the additional arm of the wye adapter during inspiration and release it during expiration to facilitate expiration. This helps the patient to expire while the equipment is connected.

Measurements

The volume of air displaced (volume per breath) and PEFR were measured in all patients, one at baseline and one at the end of the 28th session. The readings of the volume of air displaced and PEFR that were taken at the first session after the training session were considered as the baseline reading, which was the average of the best three readings. The other reading was taken at the end of the 28th session.

Patient-specific data encompassing demographic information, such as age, sex, the reason for tracheostomy, and MRC score, were recorded. Detailed documentation of the spirometry exercises included information regarding the frequency of sessions conducted per day, the number of breaths executed within each session, the volume of air displaced during each breath (volume per breath), and measurements of the PEFR obtained both before and after the spirometry test.

Statistical analysis

The patient’s demographic data were represented as frequency and percentage. The paired t-test was performed to compare the volume of air displaced and PEFR at the baseline and after 28 sessions of incentive spirometry, which followed a normal distribution. Data were analyzed using the statistical package SPSS 26.0 (IBM Corp., Armonk, NY).

## Results

Overall, 30 tracheostomy patients were included in the present study. The mean age of the patients was 42.3 ± 13.7 years. The gender-wise distribution of the patients reported that out of the total 30 patients, 18 (60%) patients were males and 12 (40%) patients were females. Table 1 displays the patient profile encompassing a study of 30 patients.

**Table 1:** Distribution of tracheostomy patients according to various parameters studied MRC = Medical Research Council.

Sr. No.	Parameter	Number of patients
1.	Reasons for tracheostomy	
	A. Respiratory failure	15
	B. Neurological condition	6
	C. Other	9
2.	MRC scale score at the start of the session	
	A. 48-50	2
	B. 51-55	10
	C. 56-60	18
3.	Details of the volume-oriented incentive spirometer protocol parameters	
	A. Number of training sessions required	
	16 patients	3
	14 patients	4
	B. Number of sessions per day	4
	C. Total number of sessions	28
4.	Details of the discomfort/complications observed following usage of the volume-oriented incentive spirometer	
	A. Pain at the tracheostomy site	4
	B. Transient hypotension during exercise	3
	C. Abdominal pain during exercise	1
	D. None	22

The pre- and post-spirometry intervention results of parameters involving lung volume and PEFR are demonstrated in Table 2. The volume of air displaced, i.e., volume per breath, significantly increased from a pre-test mean of 400 ± 124.6 mL to a post-test mean of 950 ± 330.9 mL, with a p-value of <0.001 demonstrating statistically significant results. Similarly, the PEFR demonstrated a significant improvement, increasing from a pre-test mean of 92.3 ± 26.2 L/min to a post-test mean of 134.7 ± 63.3 L/min, with a p-value of <0.001 reporting statistically significant results.

**Table 2:** Pre- and post-spirometry intervention results PEFR = peak expiratory flow rate.

Parameters	Spirometry intervention		Mean difference (95% confidence interval)	P-value
	Pre	Post		
Volume (volume per breath, mL)	400 ± 124.6	950 ± 330.9	-550.0 (-655.2, -444.8)	<0.001
PEFR (L/min)	92.3 ± 26.2	134.7 ± 63.3	-42.3 (-58.4, -26.3)	<0.001

## Discussion

The volume of air displaced in the spirometer and PEFR improved with volume-oriented incentive spirometry in patients with a tracheostomy tube and is feasible and effective in such patients. The mean volume of air displaced (lung volume) and mean PEFR at the end of seven days were 950 ± 330.9 mL and 134.7 ± 63.3 L/min, respectively. Patients exhibited an overall positive response and adaptability to the incentive spirometry device, indicating good tolerance without significant discomfort or adverse effects. A study by Malhotra et al. showed the feasibility of performing incentive spirometry after modification of the mouthpiece that adapts to the tracheostomy tube [3]. Similarly, Goldstein et al. demonstrated the usefulness of volume-oriented spirometry using modified connectors that suit patients with tracheostomy tubes. The study also demonstrated the feasibility of a modified spirometer in elective postoperative patients in the immediate postoperative period and reported that it is feasible to use incentive spirometry after modification in patients with tracheostomy tubes with minimal discomfort and complications, which increased the interest in using spirometry in such populations [4]. The above-mentioned studies concluded that incentive spirometry was well-tolerated and safe for patients with tracheostomy. However, these studies have not addressed whether the use of such a device after the recommended session of spirometry is effective in improving lung function, increasing the volume of air displaced with patient effort, or measuring PEFR, improving activities of daily living, which are commonly used to assess the improvement of respiratory function.

A study by Goldstein et al. assessed the effectiveness of an adapted incentive spirometer in 10 tracheostomy patients, particularly those who had undergone resections in the paranasal sinuses, oral cavity, and larynx. The spirometry was initiated at an average of 1.6 days postoperatively, with 3.3 sessions per day and 6.8 breaths per session [4]. In contrast, in the present study, all patients underwent 10 breaths per session and 28 sessions in seven days. This is in line with the recommendations of the American Association for Respiratory Care [21]. Moreover, the present study implemented initial training sessions for patients to learn the technique to get the desired results and studied the improvement in volume displacement at the end of the spirometry session, which showed an average improvement of 550 mL at the end of 28 sessions in comparison to the study by Goldstein et al. where the authors studied the feasibility of device and measured the average displacement of air volume in spirometer but did not studied overall effectiveness of the spirometry [4]. The present study involved a diverse population who were recovering from varied lung and neuromuscular pathologies rather than including only elective postoperative patients.

The PEFR serves as a crucial metric in respiratory medicine that helps to understand the functioning of the lungs, airways, and neuromuscular functions [18,20]. Hence, monitoring PEFR gives information on the functioning of the respiratory system. However, previous studies were conducted on patients without tracheostomy and patients demonstrating normal breathing patterns [24,25]. The literature lacks information regarding PEFR measurement in patients with tracheostomy tubes reporting a knowledge gap [26,27]. This knowledge gap is bridged in the present study reporting PEFR to be feasible and improved in all patients with an average increase of 42 L/min at the end of 28 sessions. Hence, the modified measurements of PEFR may guide in monitoring respiratory function even in patients with tracheostomy tubes [26,27].

Additionally, in the present study, it was noticed that few patients experienced pain at the tracheostomy tube site and abdomen only during the initial sessions of spirometry, which reduced over time. Moreover, a transient drop in blood pressure was observed in three patients that improved immediately at the end of the session and did not require any interventions. The study did not report any symptoms of tracheitis, wound breakdown, or clinical atelectasis, as observed in the study by Goldstein et al. [4]. This may be because the patients were evaluated in the immediate postoperative period in their study compared to the population involved in the present study.

The strength of the present study involved the utilization of modification of a volume-oriented incentive spirometry device for patients with a tracheostomy tube. The modifications are easy to do, widely available, and easy to practice. Additionally, the measurement of PEFR in patients with cuffed tracheotomy tubes by using a connector was evaluated, which was found to be feasible and measurable with ease. Moreover, the present study involved a diverse population in which the majority were recovering from critical illness rather than including only elective postoperative patients. The study presented certain limitations, which involved a single-center study and a small sample size.

## Conclusions

The present study assessed the impact of volume-oriented incentive spirometry on lung volume and PEFR in tracheostomy patients and concluded that the volume-oriented incentive spirometer improves the volume of air displacement that is lung volume and PEFR in patients with tracheostomy tubes. The volume-oriented incentive spirometry is a specialized device designed to facilitate maximal inspiration, enhance lung function, and promote deep breathing.

Hence, spirometry and measurement of PEFR are feasible and effective parameters in monitoring functions of the respiratory system in patients with tracheostomy tubes implementing an opportunity to use spirometry in patients with tracheostomy tubes in the future for which further studies may prove its beneficial impact along with better modification of the device.

## References

[1] Bartlett RH, Gazzaniga AB, Geraghty TR (1973). Respiratory maneuvers to prevent postoperative pulmonary complications. A critical review. JAMA.

[2] Rock P, Rich PB (2003). Postoperative pulmonary complications. Curr Opin Anaesthesiol.

[3] Malhotra N, Malhotra P, Verma D (2007). Incentive spirometry in tracheostomized patients. J Anaesthesiol Clin Pharmacol.

[4] Goldstein GH, Iloreta AM, Ojo B, Malkin BD (2012). Incentive spirometry for the tracheostomy patient. Otolaryngol Head Neck Surg.

[5] Nair A, Alaparthi GK, Krishnan S, Rai S, Anand R, Acharya V, Acharya P (2019). Comparison of diaphragmatic stretch technique and manual diaphragm release technique on diaphragmatic excursion in chronic obstructive pulmonary disease: a randomized crossover trial. Pulm Med.

[6] Lawrence VA, Hilsenbeck SG, Mulrow CD, Dhanda R, Sapp J, Page CP (1995). Incidence and hospital stay for cardiac and pulmonary complications after abdominal surgery. J Gen Intern Med.

[7] Wake M, Stansbie M, Thompson H (1991). Spontaneous perforation of the pharynx/esophagus. Ear Nose Throat J.

[8] Wang AS, Armstrong EJ, Armstrong AW (2013). Corticosteroids and wound healing: clinical considerations in the perioperative period. Am J Surg.

[9] Pasquina P, Tramèr MR, Granier JM, Walder B (2006). Respiratory physiotherapy to prevent pulmonary complications after abdominal surgery: a systematic review. Chest.

[10] Hristara-Papadopoulou A, Tsanakas J, Diomou G, Papadopoulou O (2008). Current devices of respiratory physiotherapy. Hippokratia.

[11] Thomas JA, McIntosh JM (1994). Are incentive spirometry, intermittent positive pressure breathing, and deep breathing exercises effective in the prevention of postoperative pulmonary complications after upper abdominal surgery? A systematic overview and meta-analysis. Phys Ther.

[12] Bloria SD, Bloria P, Luthra A, Kataria K (2019). Using endotracheal tube as an adaptor to provide incentive spirometry to tracheostomised patients. Indian J Anaesth.

[13] Overend TJ, Anderson CM, Lucy SD, Bhatia C, Jonsson BI, Timmermans C (2001). The effect of incentive spirometry on postoperative pulmonary complications: a systematic review. Chest.

[14] Kim SW, Kang HH, Kang JY, Kim SK, Lee BY, Lee SH, Moon HS (2014). A case of pneumomediastinum and parapneumonic effusions following pharyngeal perforation caused by shouting. Yonsei Med J.

[15] Roh JL, Park CI (2008). Spontaneous pharyngeal perforation after forceful vomiting: the difference from classic Boerhaave’s syndrome. Clin Exp Otorhinolaryngol.

[16] Garg R, Dutta K (2016). Incentive spirometry through tracheostomy stoma. Anaesth Intensive Care.

[17] Guimarães MM, El Dib R, Smith AF, Matos D (2009). Incentive spirometry for prevention of postoperative pulmonary complications in upper abdominal surgery. Cochrane Database Syst Rev.

[18] Bach JR, Saporito LR (1996). Criteria for extubation and tracheostomy tube removal for patients with ventilatory failure. A different approach to weaning. Chest.

[19] Lawrence VA, Cornell JE, Smetana GW (2006). Strategies to reduce postoperative pulmonary complications after noncardiothoracic surgery: systematic review for the American College of Physicians. Ann Intern Med.

[20] Antunes BO, de Souza HC, Gianinis HH, Passarelli-Amaro RC, Tambascio J, Gastaldi AC (2016). Peak expiratory flow in healthy, young, non-active subjects in seated, supine, and prone postures. Physiother Theory Pract.

[21] American Association for Respiratory Care (1991). AARC (American Association for Respiratory Care) clinical practice guideline. Incentive spirometry. Respir Care.

[22] Tan AK (1995). Incentive spirometry for tracheostomy and laryngectomy patients. J Otolaryngol.

[23] Yamaguti WP, Sakamoto ET, Panazzolo D, Peixoto Cda C, Cerri GG, Albuquerque AL (2010). Diaphragmatic mobility in healthy subjects during incentive spirometry with a flow-oriented device and with a volume-oriented device. J Bras Pneumol.

[24] Ruffin R (2004). Peak expiratory flow (PEF) monitoring. Thorax.

[25] Tomich GM, França DC, Diório AC, Britto RR, Sampaio RF, Parreira VF (2007). Breathing pattern, thoracoabdominal motion and muscular activity during three breathing exercises. Braz J Med Biol Res.

[26] Cassidy MR, Rosenkranz P, McCabe K, Rosen JE, McAneny D (2013). I COUGH: reducing postoperative pulmonary complications with a multidisciplinary patient care program. JAMA Surg.

[27] Parreira VF, Tomich GM, Britto RR, Sampaio RF (2005). Assessment of tidal volume and thoracoabdominal motion using volume and flow-oriented incentive spirometers in healthy subjects. Braz J Med Biol Res.

